# A Manual of Auscultation and Percussion

**Published:** 1849-11

**Authors:** 


### ARTICLE V.

*A Manual of Auscultation and Percussion.*—By M. Barth, Agrègè to the Faculty of Medicine of Paris, etc., and M. Henry Roger, Physician to the *Bureau Centrale* of the Parisian Hospitals, etc. Translated, with Additions, By Francis G. Smith, M.D., Lecturer on Physiology in the Philadelphia Association for Medical Instruction; one of the Editors of the Medical Examiner, etc., etc. Second Edition. Philadelphia: Lindsay & Blakiston. 1849. pp. 167.—(From the Publishers. For sale by S. C. Griggs & Co.)

This is a neatly printed duodecimo volume of 167 pages; and the first thing that strikes the attention of the reader, is

*Announcement of Jefferson Medical College for 1849-50.

the number of names that stand on the title page to father so small a book. The first sixty pages are devoted to Auscultation of the Respiratory Organs. Nearly every thing of interest in the whole is condensed, however, into the last ten pages, made up of tables taken almost entire from the work of Dr. Walshe. The general directions and explanations of the various sounds are concise, and for the most part judicious. But there are some paragraphs, which though correct enough in part, are nevertheless well calculated to produce an injurious impression on the mind of the student. Thus on page 44 we read as follows:

"The *Cavernous Voice*, like the *the Cavernous Souffle*, indicates the existence of an elliptical dilatation of the bronchi, or a tubercular, purulent, apoplectic, or gangrenous excavation.--- From the rareness of these elliptical bronchial dilatations and pulmonary excavations, independent of phthisis, compared with the frequency of cavities in phthisical subjects, we may conclude that, nine times out of ten, cavernous voice indicates tubercular excavation."

The frequency with which such numerical statements occur as are found in the last paragraph, are well calculated to beget in the mind of the student a false mode of diagnosticating disease. It leads him to take for granted, that wherever he finds *cavernous voice*, there is phthisis; while in truth, the comparative frequency of tubercular excavations is of no value whatever in determining the nature of any given disease. For instance, a patient presents himself, and on applying the ear to the chest, we detect a cavernous voice, we thereby simply know a cavity exits; but the fact that tuberculous cavities exist ten times more frequently than any other, does nothing towards proving that the particular cavity here discovered is of that nature. The physical sign proves the existence of the cavity and nothing else. The nature of the cavity must always be determined by other symptoms and the history of each case. Hence, when the authors say, as on page 35, that, "in consequence of the comparative frequency of *bronchial catarrhs*, and the comparative infrequency of the other morbid condition, in which *sonorousness* or *sibilance* manifest themselves, the *sonorous râle* announces *almost certainly* an *inflammation* or catarrhal condition of the bronchi;" they assert what is not strictly true of any given case. In the paragraph preceding the one we have quoted, we are told that this sonorous râle "may be heard in a *great many diseases*, such as inflammations or catarrhs of the bronchi, whether acute or chronic, in pulmonary emphysema, and in the compression of the air tubes by tumours," to which might be added *asthma*, and some other conditions. How then can this râle alone "*almost certainly*" determine the case to be inflammation? Admit that bronchial inflammation occurs ninety-nine times, to that of asthma, emphysema or tumours once; it does nothing towards proving that the particular case in the hands of the practitioner is not the hundredth one, i. e. asthma or some of the other affections named. Consequently it is just as necessary to examine minutely the history and signs of every case by itself, before presuming on its nature, as though emphysema or asthma were either of them as frequent as catarrh.

It is such statements as we have quoted, that induce many to rely too exclusively on certain physical signs, without connecting them carefully with the whole history of the case.—And hence they are so frequently led into error, that others are induced to undervalue physical or auscultatory signs altogether. The next 20 or 30 pages are devoted to auscultation of circulatory organs, the head, abdomen, pelvis and extremities; and all the rest of the book to percussion. The notes added by the translator are numerous and valuable; and on the whole, it is a convenient and useful little manual, both for the student and the practitioner. N.S.D.

